# Genetic diversity and evolution of endogenous pararetroviruses across *Solanaceae*: How farming systems drive dynamic tomato EPRVS changes under salt stress

**DOI:** 10.3389/fpls.2026.1702837

**Published:** 2026-01-30

**Authors:** Nadia Zitouna, Hela Sakka, Mohamed Taieb Bouteraa, Marwa Mehrez, Tarek Slatni, Hatem Fakhfakh, Karim Ben Hamed, Faten Gorsane

**Affiliations:** 1 Laboratory of Molecular Genetics, Immunology and Biotechnology, Faculty of Sciences of Tunis, University of Tunis El Manar, Tunis, Tunisia; 2 Faculty of Sciences of Bizerte, University of Carthage, Tunis, Tunisia; 3 Laboratory of Extremophile Plants, Centre of Biotechnology of Borj Cedria (CBBC), Hammam-Lif, Tunisia; 4 Faculty of Sciences of Tunis, University of Tunis El Manar, Tunis, Tunisia; 5 Manouba School of Engineering, University of Manouba, Manouba, Tunisia

**Keywords:** diversity, EPRVs, evolution, farming system, salinity, Solanaceae, tomato

## Abstract

**Introduction:**

Endogenous pararetroviral sequences (EPRVs) expand plant genome plasticity and may serve as reservoirs for adaptive evolution. Most have undergone rearrangements and mutations, leaving them largely inactive. While their integration is generally neutral, biotic and abiotic stresses can occasionally reactivate EPRVs, triggering spontaneous infections. Plants counter this by suppressing expression through sequence degeneration, fragmentation, and epigenetic silencing mechanisms such as DNA methylation.

**Methods:**

EPRVs were detected using PCR across the Solanaceae family. Genomic segments were characterized through PCR amplification, sequencing, and analyses of genetic diversity and evolutionary parameters. Two salt-contrasting tomato genotypes were cultivated under saline conditions using two farming strategies, including monocropping and intercropping with halophyte plants. The distribution of EPRVs was performed using quantitative PCR, and the methylation status was assessed through bisulfite treatment followed by PCR amplification, cloning and sequencing.

**Results:**

A Comparative analysis of genetic diversity revealed differential variability in the intergenic (IGR) region and the capsid–movement protein junction (CP-MP), likely reflecting distinct selective constraints. Haplotype networks inferred from the sequences, along with neutrality tests and mismatch distributions, indicated the occurrence of regulatory bottleneck-expansion cycles in the IGR, contrasted by signatures of purifying selection at the CP-MP junction. Quantitative PCR and bisulfite-PCR sequencing of the IGR region revealed that both the copy number of EPRVs and the level of cytosine methylation are differentially modulated in response to the cropping strategy.

**Discussion:**

These findings provide new insights into the dynamic role of EPRVs in shaping plant responses to salt stress, whereas the intercropping approach emerged as an effective farming strategy for mitigating the adverse effects of salinity stress.

## 1 Introduction

Endogenous pararetroviruses, EPRVS, members of the family *Caulimoviridae*, possess a circular double-stranded DNA genome and replicate through an RNA intermediate and are widely distributed across the plant kingdom (Sharma et al., 2020). In addition to being present episomally, these pararetroviral elements have been found within the genomes of several plant species (Staginnus and Richert-Pöggeler, 2006), including tomato (De Tomás and Vicient, 2022; Staginnus et al., 2007), rice (Chen and Kishima, 2016), tobacco (Mette et al., 2002), *Petunia hybrida* (Richert-Pöggeler and Shepherd, 1997) and banana (Chabannes et al., 2021). These elements often exhibit rearrangements and fragmentation and their integration into the plant genome is believed to occur through illegitimate recombination during the host’s DNA repair processes, allowing for their long-term retention in the plant genome (Richert-Pöggeler et al., 2021).

Most EPRVs consist of non-functional, linear viral DNA fragments incapable of forming episomal particles (Yu et al., 2019). Nonetheless, specific stress conditions or breeding interventions can trigger the reactivation of certain integrants, resulting in the reconstitution of infectious episomal forms and potential re-emergence of exogenous infections (Geering et al., 2005; Richert-Pöggeler et al., 2003; Schmidt et al., 2021).

In the genomes of angiosperms, gymnosperms, and ferns, retroelement-derived repeats can accumulate to high copy numbers and appear to share sequence homology with retroviruses and long terminal repeat (LTR) retrotransposons (Quadrana and Henderson, 2025). They commonly encode conserved enzymatic domains, including reverse transcriptase (RT), aspartate protease (PR) and RNase H (RH), along with non-enzymatic proteins such as the movement protein (MP) and the coat protein (CP). These elements are predominantly enriched in heterochromatic regions, especially AT-rich and TA dinucleotide-rich domains, and are often found adjacent to other retroelements and transposons (Schmidt et al., 2021). Unlike classical retrotransposons, EPRVs lack flanking LTRs, distinguishing them as a unique subclass of transposable elements (Jakowitsch et al., 1999).

Transposable elements (TEs) represent a dominant component of most eukaryotic genomes, including those of plants and their abundance varies substantially not only across different plant species but also among individuals within a species (Cahn et al., 2016; Domínguez et al., 2020). In *Arabidopsis thaliana*, TEs constitute approximately 21% of the genome while many other plant genomes exhibit significantly higher TE proportions, about 40% in rice, 60% in tomato, 80% in wheat, and up to 85% in maize (Jouffroy et al., 2016; Oliver et al., 2013; Quesneville, 2020). This discrepancy is largely due to the high prevalence of retrotransposons, which dominate in large plant genomes such as those of tomato (Deneweth et al., 2022). EPRVs may have been domesticated as TEs and stably integrated into host plant genomes through the evolutionary process (Yu et al., 2019). In the genus *Petuvirus* within the *Caulimoviridae* family, residues of an integrase-like domain along with quasi long-terminal repeats have been identified (Richert-Pöggeler and Shepherd, 1997, 2003), implying that some ancestral components may have been eliminated during the evolutionary development of caulimoviruses.

The integration events can occur through multiple pathways including tandem or nested insertions, ectopic recombination between non-homologous loci (Bombarely et al., 2016), reverse transcription-driven integration (Bennetzen, 2002), and unequal crossing over during meiotic prophase of tandem arrays (Gayral et al., 2008). Once integrated, these elements are heritably maintained and transmitted across generations like native host genes. During evolution, most EPRVs have undergone extensive rearrangements and accumulated mutations, rendering them functionally inactive. While the integration of EPRVs sequences into plant genomes is generally considered neutral, certain biotic and abiotic constraints can reactivate these elements, potentially leading to spontaneous infections (Valli et al., 2023). Evidence suggests that EPRVs do not always remain transcriptionally inert; their activity is typically suppressed by host silencing mechanisms, resulting in progressively fragmented or degenerated sequences. Nonetheless, integrated segments from banana streak virus (BSV) (Gayral et al., 2008), tobacco vein clearing virus (TVCV), and petunia vein clearing virus (PVCV) (Kuriyama et al., 2022) have been shown to reactivate under abiotic stress. This dynamic host genome–EPRVs interaction may culminate in the formation of viral particles and expression of infection-like symptoms. Reactivation probably requires the transcription of closely arranged integrants or the recombination of fragmented sequences, which eventually results in the formation of episomal copies. These mechanisms result in the production of virus particles and the emergence of symptoms related to viral infections (Lisch, 2009). To limit the activation and potential propagation of EPRVs, plants often suppress their expression through sequence degeneration and fragmentation, rendering the transcription of full-length viral components ineffective. In addition, epigenetic mechanisms, such as DNA methylation and histone modification, contribute to the long-term silencing of EPRVs (Dohm et al., 2014; Zakrzewski et al., 2017; Richert-Pöggeler et al., 2021).

These containment strategies may involve genomewide amplification followed by integration into heterochromatic regions for structural stabilization and transcriptional repression. Such regulatory controls help mitigate potential EPRV-induced genomic instability and viral reactivation (Schmidt et al., 2021).

In addition, it is hypothesized that the incidental genomic incorporation of EPRVs confers a form of immune memory against related pathogens, although direct evidence remains limited (Chen and Kishima, 2016; Richert-Pöggeler et al., 2021). EPRVs seem to contribute to antiviral defense within their host by mediating transcriptional gene silencing (TGS) and post-transcriptional gene silencing (PTGS), primarily via RNA-directed DNA methylation and RNA interference pathways (Kuriyama et al., 2020). Solanum species have been used as model systems to investigate EPRV composition and dynamics (Hosmani et al., 2019; Hardigan et al., 2017; Tomato Genome Sequencing Consortium, 2014). Recent studies suggest that chromosomal regions derived from EPRVs may act as sources of broad-spectrum, homology-dependent silencing, potentially encoding a heritable record of prior viral exposures (Valli et al., 2023). The presence of naturally occurring inverted repeats in EPRV loci may represent early steps in the evolution of sequence-based immunological memory.

The rapid expansion of the global human population has heightened the demand for food resources. However, agricultural productivity faces growing challenges due to various biotic and abiotic stressors, including drought and saline conditions (Tkemaladze, 2025), which significantly hinder food supply sustainability. Among these factors, salt stress stands out as a major abiotic threat, severely affecting plant development and yield (Afzal et al., 2023). The escalation of soil salinity worldwide has led to substantial reductions in crop production, adversely affecting plant health and limiting agricultural efficiency (AbuQamar et al., 2024). In addition, such challenges have contributed to the rapid spread of saline soils, decreasing the level of cultivable land and adversely affecting agricultural production (Tibesigwa et al., 2025). Utilizing salt-tolerant plants to address soil and water salinity presents a cost-effective and efficient phytoremediation approach, enhancing agricultural productivity in salt-affected areas. Numerous halophyte species exhibit a remarkable ability to absorb and store salt within their tissues, making them valuable for mitigating salinity-related challenges (Simpson et al., 2018; Wang et al., 2022). Increasing research focuses on integrating halophytes into intercropping or sequential cropping systems to facilitate soil desalination (Ben Hamed et al., 2021, Slatni et al., 2024). The practice of intercropping with halophytes has shown promising results in enhancing the growth and yield of agriculturally important plants (Jurado et al., 2024; Slatni et al., 2024; Sogoni et al., 2025). Graifenberg et al. (2003) observed that the presence of the halophyte, *Suaeda salsa*, mitigated the negative effects of salt and improved tomato plant performance in terms of shoot dry weight and fruit production. Hence, mixing tomato with the halophyte *Portulaca oleracea* resulted in increased plant growth and fruit production and alleviates the impact of salinity on tomato yield (Zuccarini, 2008). Additionally, reports indicated that the intercropping system combining cotton and halophytes exhibited superior performance in salt removal and crop productivity compared to the monoculture system (Liang and She, 2021). Adopting a tomato and halophyte cropping system seems a powerful opportunity to significantly improve the sustainability of salt-sensitive crops in areas affected by salinity (Jurado et al., 2024). However, such a strategy has to be managed correctly to avoid competition among species and optimize natural resource utilization (Maitra et al., 2021).

Tomato (*Solanum lycopersicum* L.), one of the most important vegetable crops cultivated in the Mediterranean region, is moderately sensitive to salinity (Wang et al., 2023). Under salt stress conditions, tomato plants exhibit reduced nutrient use efficiency, which poses a challenge to sustaining high yield and fruit quality (Patanè et al., 2011; Wang et al., 2020). Understanding the adverse effect of salt stress on tomato crops is crucial for developing and implementing effective practices to meet market demands. To address this challenge, our study focuses on the dynamic role of EPRVs as key biomarkers of salt stress adaptation in contrasting tomato genotypes. EPRVs were identified across Solanaceae species, including tomato, eggplant, and tobacco. We specifically analysed genetic diversity and evolutionary scenario within two pararetroviral segments corresponding to the intergenic region (IGR) and the capsid–movement protein junction (CP-MP). Concomitantly, we evaluated the influence of cropping systems on EPRV dynamics by comparing conventional monocropping against intercropping involving halophyte species under saline conditions. We further examined EPRV distribution and the methylation status of the IGR region. Our findings highlight the regulatory flexibility of EPRVs and demonstrate the agronomic benefit of intercropping as a promising strategy for enhancing crop resilience and promoting sustainable agriculture.

## 2 Materials and methods

### 2.1 Plant material and greenhouse cultivation

Plant material correspond to (i) *Solanum lycopersicum:* Coeur de boeuf (Cbf), Sabra (Sb), Saint-pierre (Sp), Marmade VR (Mar), Rio grande (Rio), Merveille des marchés (Mdm); (ii) *Capsicum annuum:* Marconi rosso (Mr), Corno di toro rosso (Cdt), Picante rosso (Pr); (iii) *Solanum melongena:* Bellezza near (Bn), Mezza lunga violetta (Ml)) and (iiii) *Nicotiana tabacum* Xanthi (N). These cultivars are commonly used by Tunisian farmers and their corresponding seeds were provided by the Laboratory of seeds and plant analysis (Ministry of Agriculture, TUNISIA). Seeds were disinfected using a solution of 95% ethanol and 0.1% tween and then planted in 500 mm high pots filled with a mixture of plant compost (40%), peat moss (40%), and sand (20%). The soil was enriched with NPK 20-20–20 fertilizer (pH 6.8, Terranum). 10 plants for each cultivar were watered from the bottom every 3 days. The experiments took place in an environmentally controlled greenhouse, located at the Faculty of Sciences of Tunis (GPS Coordinates 36.8125°N, 10.1387°E), Tunisia. The pots were arranged randomly under controlled conditions (40-60% relative humidity; 8/16 h dark/light cycle (100 μmol/m^2^.s) with a day/night temperature of 25°C/18°C.

### 2.2 DNA extraction and EPRVs amplification

Genomic DNA was isolated from fresh leaves using the Analytik Jena Extraction Kit following the manufacturer’s instructions. The kit corresponds to a DNA extraction method specifically designed to isolate genomic DNA and actively excludes the small, circular, episomal forms. DNA quantification was performed with an ND-1000 spectrophotometer (Nanodrop Technologies, USA). PCR amplification was performed using degenerate primers to target the pararetroviral CP-MP junction (F:5’CWTGTTAYAAYTGYGGAAARWTAGGAC3’ and R:5’TTTCWATRGGNGTATCTATTCCTTCTC3’) and the intergenic region IGR (F: 5’CWYTTAAGWTYATGAGTAGCTAWATTAATTTATTCCTG3’ and R: 5’CCTCAAMTYTGTTTAMTCCCCTAAACGG3’) (Staginnus et al., 2007). PCR reaction was performed in a final volume of 25 μl containing 100 ng of each primer, 5 μl of 10X buffer, 1 μl of dNTP (10 mM each), 1.5 μl MgCl_2_ (1.5 mM), 1U of Taq DNA polymerase (MP Biomedicals, Europe), 2 μl of extracted DNA, and sterile water. PCR amplifications were performed on a T professional TRIO system thermocycler (Biometra, Germany) using the following cycling program: One first denaturation step of 3 min at 94°C followed by 35 amplification cycles, each consisting of 30 sec at 94°C, 50 sec at 55°C, 1 min at 72°C. A final step of extension is performed at 72°C for 10 min.

### 2.3 Sequence analysis

PCR amplicon bands were extracted using the Qiagen Gel Extraction Kit and eluted in 30 μL H_2_O. Purified products were subjected to the Sanger sequencing method in an automatic sequencer Genetic Analyzer (Applied Biosystems, USA). Three amplicons, obtained from two independent experiments, were sequenced for each considered pararetroviral region. Sequences corresponding to both IGR and CP-MP were aligned and evaluated qualitatively checked using BLASTn algorithm and further edited manually using Chromas software 2.6.6 (Technelysium Pty Ltd). Multiple alignment was performed using Muscle algorithm implemented in MEGA11 software (Tamura et al., 2021).

Pairwise nucleotide sequence comparison was calculated and schematized using Sequence Demarcation Tool (SDT) version 1.2 (Muhire et al., 2014). Multiple alignment of the obtained sequences was achieved (MEGA11 software). to be analyzed with the DnaSP program version 6.0 (Rozas et al., 2017). Several parameters were taken into account as the total number of segregating sites (S), the haplotype diversity (Hd) (Nei and Tajima, 1983), and pairwise estimates of nucleotide divergence (π) (Jukes and Cantor, 1969). The haplotypic relationships between all analyzed samples were translated into a network using the software Network version 4.5.0.0 (Bandelt et al., 1999). A neutrality test was conducted to verify the neutral mutation hypothesis by using an average number of nucleotide differences (k) in the IGR and CP-MP frames and the number of segregation sites employed. Parameters as Tajima’s test (Tajima, 1989), R2, the raggedness statistic index (r), Fu’s Fs test and the mismatch analysis (MMA) were assessed. Considering that, the junction between CP and MP is a coding region, the Codon Usage Bias was established (MEGA11 software).

### 2.4 Salt scale-class classification

At the stage of four leaves, either Coeur de Boeuf (Cbf) or Sabra (Sb) genotypes were transplanted into separate plastic pots filled with a mixture of peat and sand. The salt stress regime involved applying a 50 mM NaCl solution (6 dS/m) on the first day, followed by an increase to 100 mM (12 dS/m) on the second day and finally to 150 mM (15 dS/m) on the third day. We used three biological replicates, each consisting of a pool of 10 plants arranged randomly. A set of three plants for each genotype was growing in a non-saline condition and watered with the nutrient solution serving as the experimental control (T^C^). Three weeks post-treatment, plants were evaluated for salt tolerance by comparing visual phenotypes of salt-treated genotypes against normally watered controls. Plants were evaluated and rated according to the salt scale class established by Chookhampaeng et al. (2007).

### 2.5 Farming practices and field experimental design

**Figure 1:**
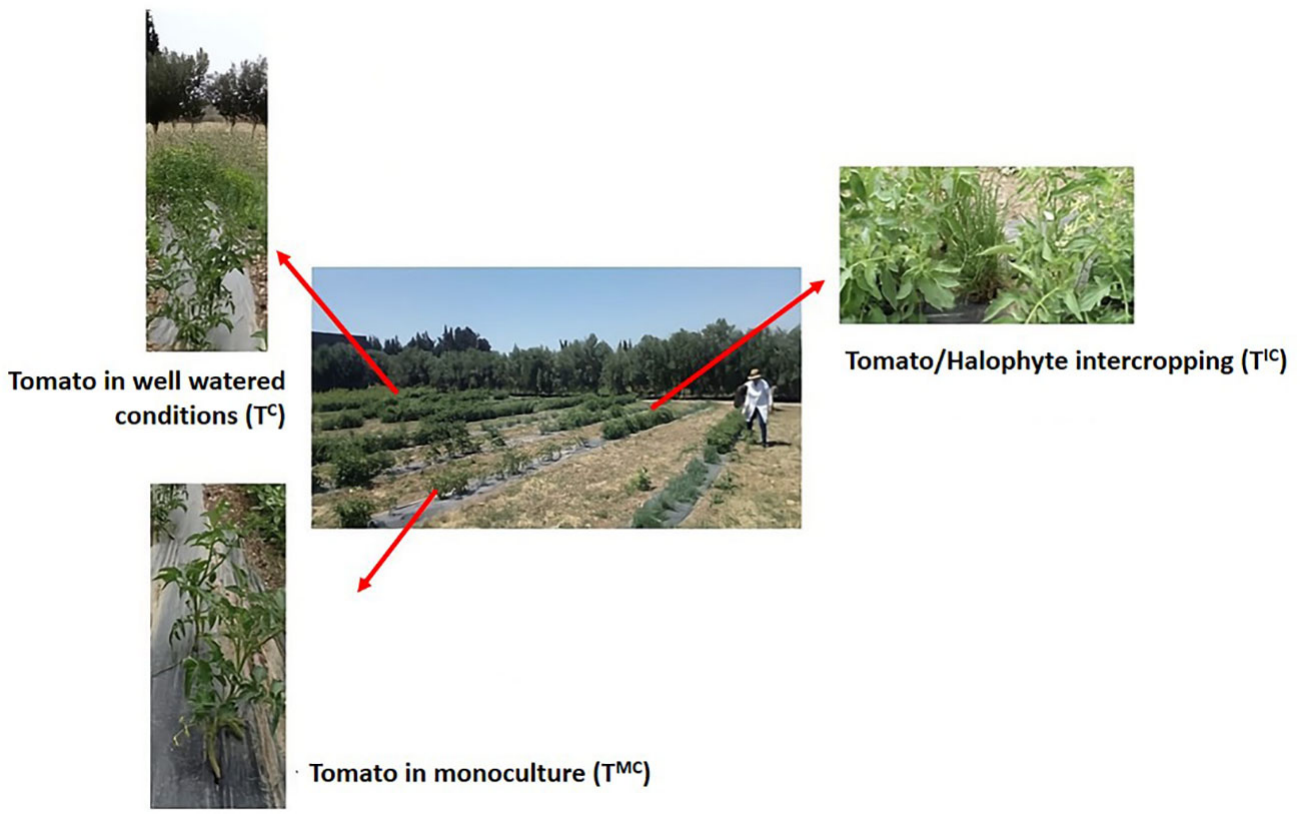
Experimental field design showing tomato plants in monoculture (T^MC^) and in intercropping with halophytes (T^IC^) alongside tomato plants under well-watered conditions (T^C^).

Farming cultures design was set up as reported by Slatni et al. (2024). Briefly, the experimental field design was organized in a randomized complete block layout with three replications and involving both contrasting tomato genotypes (**Figure 1**). Two growing schemes were addressed in this work (i) tomato in monoculture (T^MC^) and (ii) tomato cultivated alongside the halophyte *Arthrocaulon macrostachyum* L. using an intercropping approach (T^IC^). Tomato plants grown in well-watered conditions (T^C^) were considered as a control. Each experimental plot consisted of a single 6-meter row containing 15 plants with 40 cm spacing between individuals. Tomato seedlings, acquired from a local Tunisian nursery, were transplanted in mid-March alongside halophyte plants obtained from the CBBC nursery. The experiment was conducted at a field site in northeastern Tunisia’s Soliman region (N43°40’30.7”, E10°18’38.6”). Following the growth period, the top leaves of plants were meticulously collected to ensure subsequent analysis and insights.

### 2.6 EPRVs copy number quantification

Quantitative PCR (QPCR) was performed in ABI A Prism 7500 Sequence Detection System (Applied Biosystems, USA) under the following conditions: 10 min at 95 °C followed by 40 cycles of 15 sec at 95 °C, 1 min at 60 °C. The reaction was achieved using Igreen qPCR master Mix-Rox (BIOMATIK, USA). Three technical replicates were conducted for each sample. IGR Primers were used and the tomato β-actin gene as an internal gene reference (Løvdal and Lillo, 2009). The copy number was calculated based on the 2-ΔΔCt method (Livak and Schmittgen, 2001) using DataAssist™ v3.0 Software (Applied Biosystems, USA). Results were analyzed using GraphPad Software (version 6.0, CA, USA). A heat map was constructed based on Euclidean distance using DataAssist™ v3.0 Software (Applied Biosystems, USA). The mean copy number represents the median of ΔCt values obtained.

### 2.7 Bisulfite conversion, cloning and sequencing

Fresh leaves were collected 30 days post-seedlings and total genomic DNA was isolated using Analytik Jena extraction Kit. An additional step corresponding to Proteinase K treatment (20 mg/ml) (Scientific Thermofisher) was performed. 500ng of DNA was treated with sodium bisulfite (Martinez et al., 2014) using the Methylation Kit (Zymo, USA). Modified DNA was amplified by PCR using IGR1/2primers and Taq DNA polymerase (MP Biomedicals, Europe). The amplicons were purified from agarose gel and cloned using the pGEM-TEasy vector system (Promega). Three clones were selected for sequencing analysis for each cultivar. Sequencing was performed in both strands with an automated DNA sequencer ABI Prism DNA-377 apparatus (Applied Biosystems, Paris, France. The quality of bisulfite sequence data was evaluated using BioEdit. Alignment of the sequences was performed with ClustalW in MEGA11. The aligned files were processed by Cymate software to calculate the average methylation of cytosine bases in the context of symmetric CG and CHG, and asymmetric CHH (where H = A, C, or T).

## 3 Results

We explored the presence and the distribution of EPRVS sequences within a collection of plant genomes from the solanaceous family. We aimed to assess their genetic diversity and provide a more comprehensive understanding of their dynamic contribution in enabling tomato plants to withstand salt stress (**Supplementary Figure S1**).

### 3.1 Genomic identification and diversity of solanaceous EPRVs

The investigation encompassed twelve cultivars representing four predominant Solanaceae species in Tunisia: tomato (*Solanum lycopersicum*), pepper (*Capsicum annuum*), eggplant (*Solanum melongena*), and tobacco (*Nicotiana tabacum*). The integration of the EPRVs into solanaceous genomes was assessed through PCR amplifications of the intergenic region IGR and the junction between the capsid and the movement proteins CP-MP. PCR amplification products were initially assessed via agarose gel electrophoresis, revealing distinct and uniform fragment patterns of 200 bp and 600 bp for IGR and CP-MP junction regions, respectively (**Supplementary Figure S2**). The results indicate the presence of all amplicons at the expected sizes, confirming the unequivocal presence of EPRVS in all the tested host plants. Following amplicon purification, and Sanger sequencing, the resulting sequences were corrected and validated using the Chromas program. Next, these sequences were submitted to databases using the BLASTN program to confirm their reliability and to identify the genetically closest EPRV.

Sequence identities ranged from 88.8% to 98.7% in tomato, from 98% to 100% in pepper and around 98.7% in eggplant. Comparison of IGR sequences between different plant species showed very highidentity, with a minimum of 80.9% between tobacco and tomato and a maximum of 100% between tomato and eggplant, tomato and chilli and chilli and eggplant. Compared with the sequences available in the databases, the EPRV showed the highest sequence similarity with that of TVCV (tobacco vein clearing virus), an American isolate described in *Nicotiana edwardsonii* (AF190123) (**Table 1**).

**Table 1:** Sequence identities of the IGR region between solanaceous species.

Variable	Tomato	Pepper	Eggplant	Tobacco	TVCV
Tomato	88.8-98.7%	–	–	–	–
Pepper	91.5-100%	98-100%	–	–	–
Eggplant	92.2-100%	98-100%	98.7%	–	–
Tobacco	80.9-86.3%	84.9-85.7%	85.1-85.5%	100%	–
TVCV	77.1-81.4%	81.1-81.9%	81.4%	88.3%	100%

TVCV, tobacco vein clearing virus.

**Figure 2:**
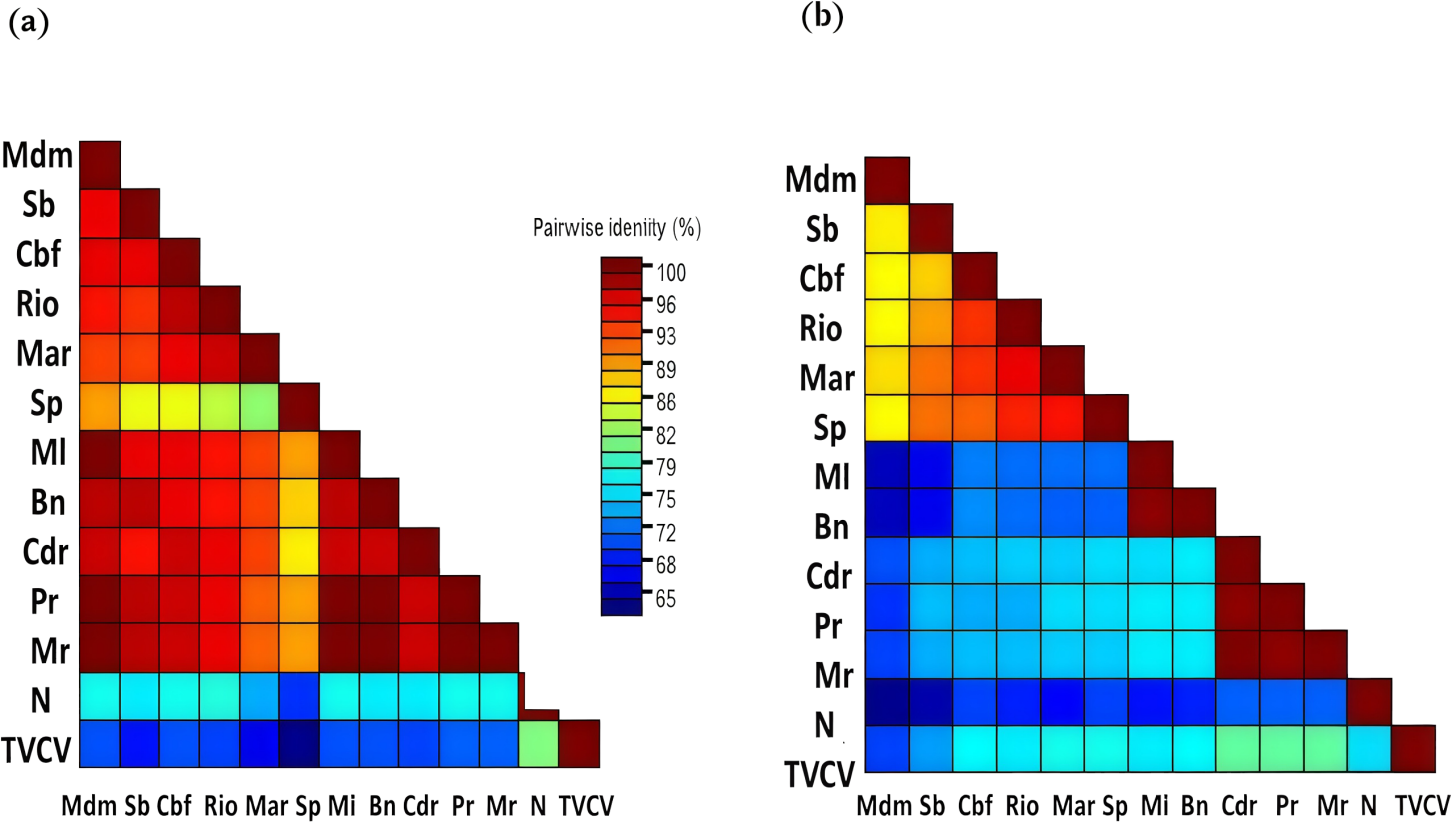
Pairwise nucleotide identity generated by SDT based on **(A)** the intergenic region IGR and **(B)** the CP-MP junction. Tomato: Coeur de boeuf (Cbf), Sabra (Sb), Saint-pierre (Sp), Marmade VR (Mar), Rio grande (Rio), Merveille des marchés (Mdm); pepper: Marconi rosso (Mr), Corno di toro rosso (Cdr), Picante rosso (Pr); eggplant: Bellezza near (Bn), Mezza lunga violetta (Ml); tobacco: Xanthi (N) and TVCV (AF190123).

The sequences amplified using the CP/MP primer pair (600 bp) revealed intra-species sequence identities ranging from 86.0% to 95.7% in tomato and from 99.2% to 99.8%, with an average of approximately 99.6%, in eggplant. Inter-species comparisons showed a minimum identity of 62.9% between tobacco and tomato and a maximum of 76.0% between eggplant and pepper, indicating species-specific divergence of this genomic region. Notably, pepper exhibited the highest homology with t tobacco vein clearing virus (TVCV), whereas tomato showed the lowest, with sequence identities ranging from 69.4% to 77.3% (**Table 2**). Afterwards, IGR and CP-MP sequences were aligned and compared to each other using the SDT program (**Figure 2**). Our results indicate that the IGR region exhibits a greater degree of conservation compared to the CP-MP region among the tested cultivars from the Solanaceae family.

**Table 2:** Sequence identities of the CP-MP junction between solanaceous species.

Variable	Tomato	Pepper	Eggplant	Tobacco	TVCV
Tomato	86-95.7%	–	–	–	–
Pepper	68.9-75.3%	99.2-99.8%	–	–	–
Eggplant	64.8-72.1%	75.4-76%	99.6%	–	–
Tobacco	62.9-69.5%	70.6-70.9%	67.7-68.3%	100%	–
TVCV	69.4-77.3%	80-80.2%	76.1-76.4%	75.4%	100%

TVCV, tobacco vein clearing virus.

### 3.2 Haplotype networks

**Figure 3:**
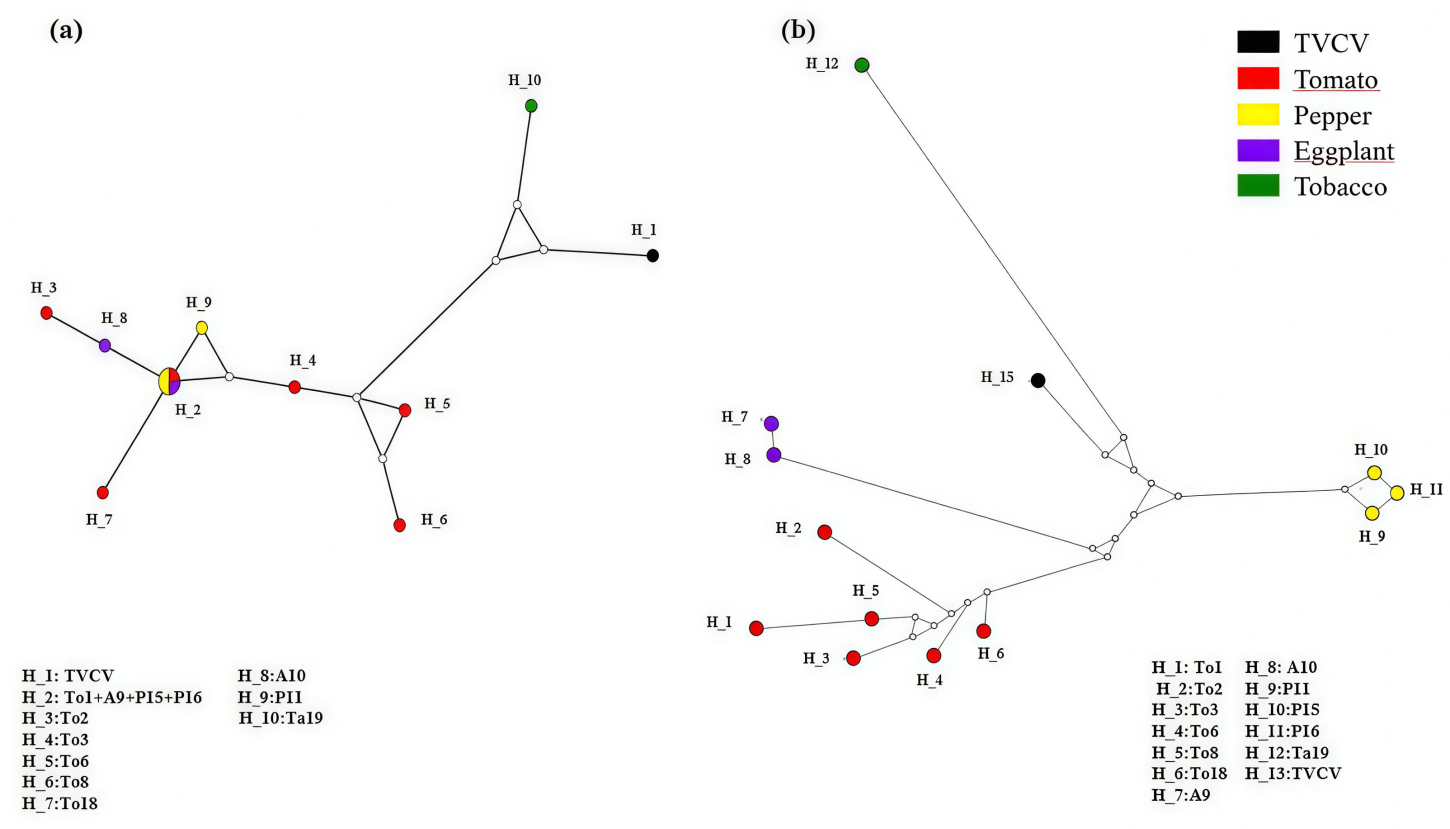
Network of the inferred haplotypes of **(A)** the IGR region and **(B)** the CP-MP junction. The size of the nodes is proportional to the haplotype frequencies; the size of the branches is proportional to the number of mutations between haplotypes; empty circles correspond to mutations. Haplotypes correspond to sequences within tomato: To1 (Mdm), To2 (Sb), To3 (Cbf), To6 (Rio), To8 (Mar), To18 (Sp); pepper P11 (Cdr), P15 (Pr), P16 (Mr); eggplant: A9 (Ml), A10 (Bn), tobacco: Ta19 (N2).

Evolutionary analysis using the haplotypic network method was assessed for both the IGR and the CP-MP Junction regions (**Figure 3**). Regarding the IGR region, the median-joining haplotype network displayed 10 haplotypes (**Figure 3A**). The H2 haplotype is the only one that includes multiple sequences, encompassing four sequences from different hosts, such as pepper (Pr and Mr) along with tomato (MdM) and eggplant (Ml). This suggests that the major haplotype H2 could represent a common ancestor. Otherwise, H10 (tobacco) and H1 (TVCV) appear to be the most divergent with long branches indicating a high mutational step. The CP-MP junction sequences fit into 13 haplotypes (**Figure 3B**). The network topology showed five assigned clades species-dependent. Indeed, distribution revealed that haplotypes are assigned into groups based on their host plant of origin. Different haplotypic groups diverge from each other through numerous mutations. Mutations changes are thus being driven by host-specific selection rather than accumulating randomly.

### 3.3 Genetic diversity

**Figure 4:**
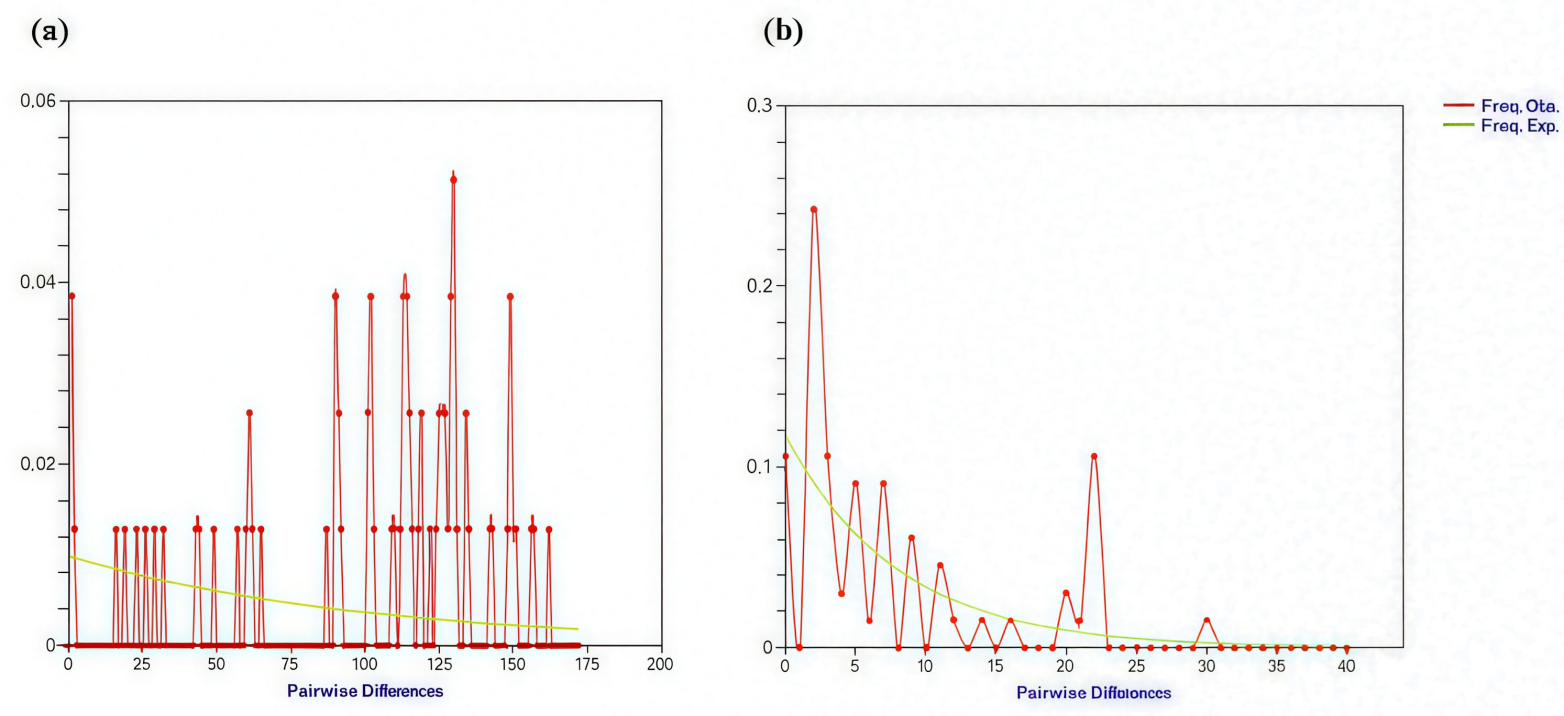
Mismatch analysis (MMA) of pairwised nucleotides on the **(A)** CP-MP region and **(B)** IGR region. Solid line: Observed pairwise differences. Dotted line: expected results of pairwise differences. Numbers of nucleotide differences between all pairs of sequences are indicated along the x-axis, and the frequency of pairs is indicated by the y-axis.

Parameters of genetic diversity were assessed among the overall sequences of both IGR and CP-MP from the involved Solanaceae species (**Table 3**). The neutrality tests for both the intergenic CP-MP and the IGR regions showed a total number of 585 and 159 sites, respectively, among which 262 and 34 corresponded to segregation sites, respectively. The historical demographic patterns were explored by two different approaches including R2 test, Fs test of Fu (1997) and the D test of Tajima (1989) as well. This genetic diversity is corroborated by positive values of the neutrality indices R2 (0.186 and 0.164), Fs (0.231 and 0.005) and r (0.031 and 0.150) and negative values of Tajima D (- 0.299 and - 1.183) for CP-MP and IGR, respectively. The average number of nucleotide differences k of 109.167 (CP-MP) and 7.500 (IGR) argued for multimodal distributions of the mismatches of pairwised nucleotides (**Figure 4**).

**Table 3:** Genetic diversity within IGR and CP-MP regions of the EPRVs.

Test	CP-MP	IGR
S, Number of total sites	585 (429 excluding gaps)	159 (149 excluding gaps)
Svs, Number of segregating sites	262	34
Hd, Haplotype diversity	1	0.894
Π, Nucleotide diversity	0.254	0.05
R2 statistic ^NT^	0.186	0.164
Tajima D ^NT^	-0.299 ^NS^	-1.183*
Fu’s Fs ^NT^	0.231	0.005
Raggedness statistic, r ^NT^	0.150	0.031
Average number of nucleotide differences, k ^NT^	109.167	7.500

^NT^, Neutrality tests; ^NS^, Not significant; *, Significant at p < 0.05.

Within both regions, the mismatch distribution displayed multimodal patterns, indicating that sequences have undergone stable population size and subdivision over long periods. When plotting the frequency distribution of nucleotide differences between possible sequence pairs, multiple peaks are observed with a great number in the CP-MP region despite the presence of one major peak. Assuming that the IGR region tends to exhibit a more likely bimodal distribution, a lower level of genetic divergence arises compared to the CP-MP.

Examining the AAs sequences in the CP-MP region, analysis revealed the occurrence of several stop codons (**Supplementary Table S1**). Furthermore, the codon usage bias for all the AAs and codons were calculated and reported. The frequencies of the stop codons including UAG, UGA, and UAA, were found to be 0.76, 1.25, and 0.99, respectively, with codon usages of 3.7, 6, and 4.8. The most frequently used codon was AGA, with a codon usage of 4.05, while UCG, GGC, and GGG were not represented in the analysis.

### 3.4 Dynamics of tomato EPRVs across farming practices

The responses of tomato plants to salt stress were explored by screening the copy number of EPRVs inserted in their genome and examining the methylation status of a pararetroviral fragment within the IGR region. To that end, we surveyed active changes in two salt-contrasting genotypes across two different farming practices (i) tomato in monocropping (T^MC^) (ii) tomato in intercropping with halophytes (T^IC^). We aimed to understand the impact of these management systems on the resilience and adaptation of tomato plants to salt stress with particular emphasis on the EPRVs dynamics.

### 3.5 Discrimination of contrasting salt-responsive tomato genotypes

Salinity regime negatively impaired tomato plants in the greenhouse and the differences among the phenotypes became more pronounced three weeks post salt stress imposition. Screening of visual symptoms was assessed according to a salt scale susceptibility based on the grading system (Chookhampaeng et al., 2007). Although neither genotype was adapted to salinity stress, a thorough screening process was implemented to categorize all tested varieties into mildly salt-tolerant and salt-sensitive categories. The Cbf genotype exhibited yellowing and slightly rolled leaves and was therefore assigned to the mildly tolerant scale class, while the Sabra genotype was considered as sensitive due to severe wilting and slow growth.

The selected Cbf and Sabra genotypes were cultivated using an outdoor phytodesalination strategy, a novel and cost-effective biological approach designed to mitigate soil salinization. Apart from monocropping (T^MC^), the strategy involves intercropping (T^IC^) which is the practice of growing tomato and halophyte crops simultaneously, so they coexist and interact with each other and the surrounding agroecosystems. These farming practices were undertaken in the field under a salt stress regime (5g/L). Upon visual screening, salinity adversely affected the phenotypes of both cultivars by slowing their growth and development. Surveying meticulously the field revealed that tomato plants (T^IC^) displayed a notable recovery, regardless of the considered cultivar. Meanwhile, the Cbf cultivarexhibited the best recovery compared to the Sabra cultivar.

### 3.6 EPRVs copy number variations

**Figure 5:**
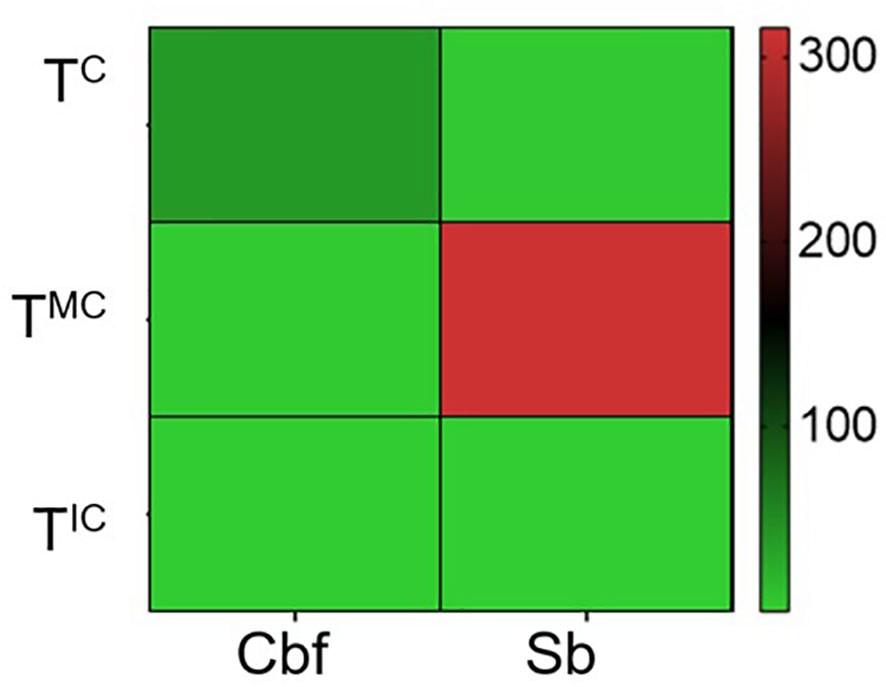
Copy number status of EPRVs in both salt-tolerant (Cbf) and sensitive (Sb) genotypes. Clustering (Average linkage, Euclidean distance) was applied to group EPRVS with similar levels. The color intensity indicates abundance level with a color scale ranging from red (high) to green (low). T^C^, tomato in well-watered conditions (control); T^MC^, tomato in monocropping; T^IC^, tomato in intercropping with halophytes, under salt stress.

The QPCR method, targeting the IGR region, was used to accurately estimate the copy number of EPRVs within the genomes of both contrasting genotypes. This DNA-based approach provides the number of integrated elements per genome regardless of their transcriptional status. The findings revealed that the abundance of EPRVs fluctuates according to the cultivation strategy (**Figure 5**).

Results showed that under the T^MC^ strategy, salt stress significantly increased the accumulation of EPRVs in the genome of the salt-sensitive Sabra genotype, whereas T^IC^ management tended to decrease this level. Regarding the salt tolerant Cbf genotype, it does not follow a similar pattern. The T^MC^ strategy did not significantly affect the abundance of EPRVs, while the slightly reduced abundance in the T^IC^ strategy was not statistically significant.

### 3.7 Dynamic methylation changes in the IGR region

**Figure 6:**
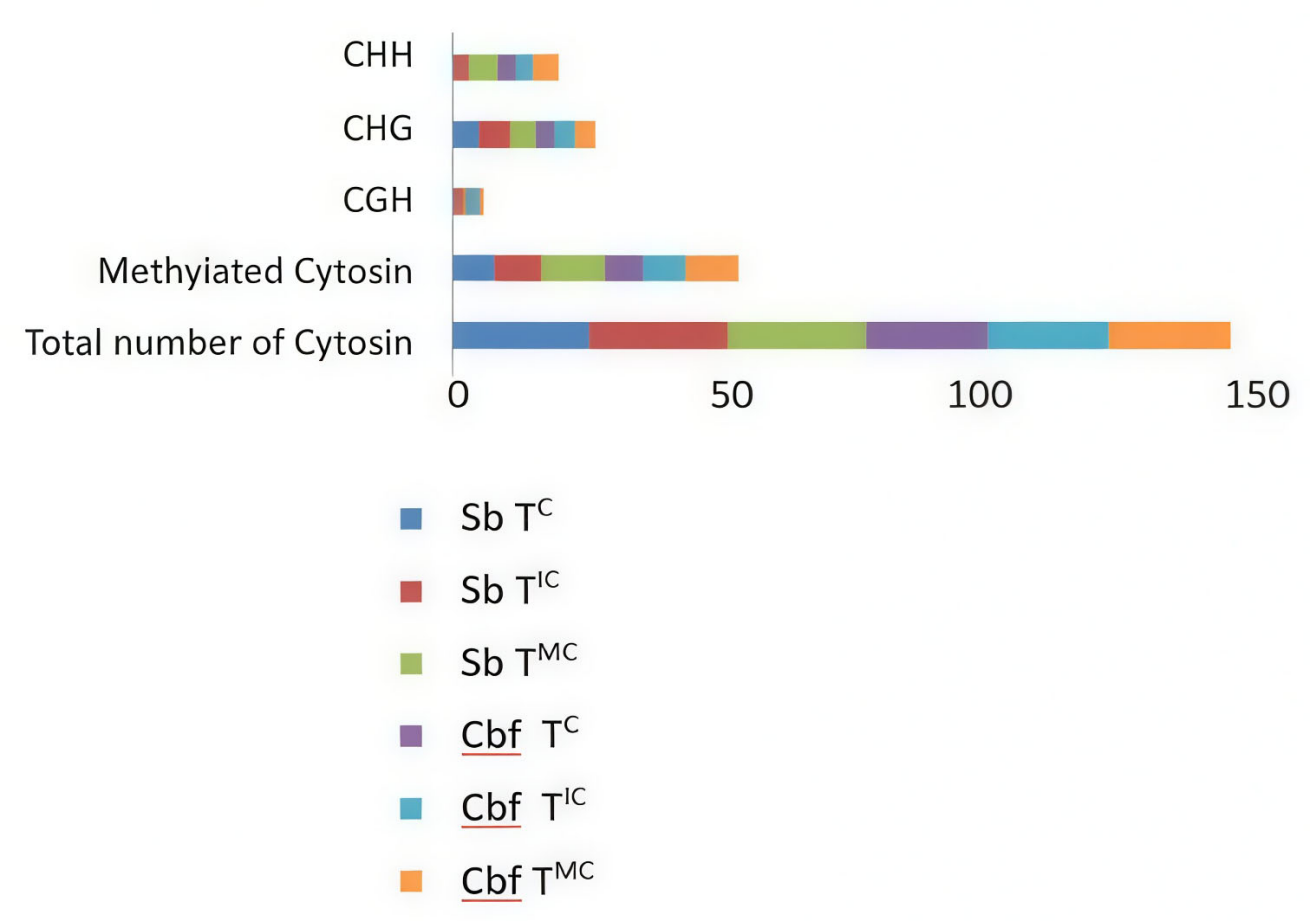
Grouped-Histogram illustrating the variation of the cytosine methylation patterns within the IGR region. T^C^, tomato in well-watered conditions (control); T^MC^, tomato in monocropping; T^IC^, tomato in intercropping with halophytes under salt stress.

To investigate whether the differential response to salinity stress is associated with alteration in cytosine methylation (mCs), we focused on the DNA methylation patterns of the IG region within both contrasting genotypes. Therefore, the DNA of tomato plants from T^MC^ and T^IC^ was extracted, bisulfite converted before PCR amplification, cloning and sequencing. Analysis of multiple clone sequence alignments was compared to those obtained from T^C^ and findings supported that the IGR corresponds to a differentially methylated region (DMR). The variation of the cytosine methylation patterns within the IGR region assessed (**Figure 6**).The total number of cytosines was 23 in Cbf and 26 in Sabra genotypes distributed in the context of symmetric CGH and CHG, and asymmetric CHH (where H = A, C, or T) islands. The bisulfite sequencing results revealed that the methylation status of the CGH sites remained unchanged regardless of the genotype or the experimental practices for the plants’ growth. However, significant differences were detected in the CHG and CHH sites, which were found to be substantially methylated.

The untreated Cbf cultivar exhibited an average mCs percentage of 30.43%, while T^MC^ and and T^IC^ strategies enhanced the cytosine methylation levels to 43.47% and 33%, respectively. In fact, upon salt treatment, T^MC^ cultivars displayed enhanced methylation levels of CHG (33.3%) and CHH (66.6%). Meanwhile, the T^IC^ maintained the level of CHH methylation while increasing CHG by 22.22%. The untreated Sabra cultivar followed the same trend of Cbf with an average mCs percentage of 30.76%, while T^MC^ and and T^IC^ displayed mCs levels of 47.43% and 32.05%, respectively. When subject to salt treatment, notable changes in the distribution of methylated CHG and CHH were observed, compared to the control. Hence, the T^MC^ cultivars increased the methylation levels of CHG (20%) and CHH (166.6%) %). In addition, the T^IC^ maintained the level of CHG methylation while slightly increased CHH methylation 16.66%.

For each genotype considered individually, T^MC^ strategy affects the methylation of CHG and CHH, while T^IC^ tends to restore the methylation status of the CHH to that of the control compared to T^MC^.

## 4 Discussion

### 4.1 EPRVs diversity patterns within the Solanaceae family

EPRVs are widely integrated into plant genomes, where their variable accumulation or elimination across hosts influences genome architecture (Richert-Pöggeler et al., 2021). While their diversity has been investigated, previous analyses were limited by the available data (Sahebi et al., 2018). The recent substantial expansion in sequenced plant genomes allowed a comprehensive screening for EPRVs and enabled a more extensive understanding of how these endogenous elements are distributed throughout plant genomes. Studies to date suggest that EPRVs are abundant in various members of the Solanaceae family and may have originated from different genera (Staginnus et al., 2007). In this study, we achieved molecular identification of EPRVs within the Solanaceae family, including cultivars of tomato, pepper, eggplant, and tobacco. To further characterize these elements, PCR amplicons corresponding to the coding CP-MP junction and the non-coding IGR were generated and sequenced. Comprehensive analysis of EPRV sequences from various host plants revealed a high degree of conservation within the IGR across all tested species. This observation aligns with prior studies reporting IGR conservation in tomato, tobacco, and potato (Staginnus et al., 2007). The nucleotide identity among isolates is recorded at a minimum of 80.9%, which notably exceeds the 80% threshold typically used to distinguish species boundaries (Guimarães et al., 2015). Pairwise comparisons of the IGR revealed over 80% nucleotide identity between the analyzed sequences and the TVCV isolate. These findings further support the classification of the Tunisian isolates within the *Solendovirus* of the *Caulimoviridae* family.

Sequence identity analysis of the CP-MP junction revealed strong conservation within individual host plant species. In contrast, interspecific comparisons of nucleotide sequences yielded identity values not exceeding 80.2%, a threshold close to the 80% criterion used to define distinct pararetroviral species (Geering and Hull, 2012; Guimarães et al., 2015).

The high rate of polymorphism observed in the CP-MP junction compared to the IGR region suggests that the CP-MP segment displays a greater genetic variability, potentially driven by weak purifying selection or increased local mutation rates.

The CP-MP region, potentially involved in functional regulation (Almeyda et al., 2014) seems to accommodate high sequence variations without compromising genomic integrity. The pronounced variability observed in this segment may reflect adaptive potential, possibly enhancing the efficient host-EPRVs plasticity and dynamics. In contrast, canonical IGRs often harbor regulatory elements essential for gene expression, leading to stronger evolutionary conservation due to functional constraints (Staginnus et al., 2007). Application of a structured coalescent framework to model purifying selection revealed that weak selection pressures can lead to elevated levels of polymorphism in certain genomic regions relative to others (Strütt et al., 2025). EPRVS within strawberry genomes were analyzed using homologous sequence searches and genome alignment approaches revealing a broad spectrum of variability (Wang et al., 2024). Comparative analysis between *Fragaria* species and closely related plant genera highlighted genus-specific differences in virus-like sequence composition, along with conserved elements shared among related genera.

The presence of stop codons within the CP-MP coding region of the EPRV appears consistent with genomic fragmentation into multiple reading frames, subsequently modified by deletion events. This observation aligns with findings by Staginnus et al. (2007), who reported that only one tomato clone retained a complete CP sequence, exhibiting 65% to 94% identity with CP fragments from other clones. Such arrangements are commonly represented among *Eucalyptus grandis*, where the Gag domain coding a structural protein essential for pararetrovirus assembly and maturation is delete (Marcon et al., 2017). Within the rice genome, certain endogenous rice tungro bacilliform Virus-like elements (eRTBVLs) exhibit an intergenic region (IGR) that appears more abundant than other coding segments (Chen et al., 2014). Additionally, endogenous PVCV-like elements (ePVCVs) identified in rice contain the MP, PR, and RT/RH domains, but lack the CP domain (Chen et al., 2017). In the Solanaceae family including Nicotiana species s, cloned protein-coding regions are either truncated or contain multiple frameshifts and stop codons, making them translationally defective. This phenomenon has also been observed in *Nicotiana* EPRVs (Teycheney and Geering, 2011; Staginnus et al., 2007). Additionally, rearranged and immuno-competent chromosomal regions, serving as heritable trans-acting markers of past infections, contribute to the persistence of latent EPRVs (Valli et al., 2023).

### 4.2 Tracing the molecular evolution of EPRVs

The inferred haplotypic networks’ topology, combined with neutral evolution analysis supports the hypothesis of EPRVs’ genome mosaic evolution, indicating that the IG and CP-MP regions are evolving under different selective regimes. Neutrality tests operate under distinct feasible conditions, primarily defined by the timing and intensity of selection. They detect selective events only within a restricted parameter range, so the observable sweeps represent only a fraction of those that actually occurred. For instance, Tajima’s D is particularly effective for identifying adaptations and selection under a wider range of parameters (Tanaka et al., 2023). The IGR regions often contain regulatory elements (promoters, enhancers, RNA secondary structures) that are functionally constrained. In the haplotypic network inferred from IGR sequences, the lack of host-specific clustering suggests these sequences may be under similar selective pressures across different plant hosts, maintaining their function regardless of the host environment. This pattern argues for a purifying selection acting on functional elements to remove deleterious variants, creating an excess of rare mutations and maintaining functional conservation across different plant hosts. Population expansion is likely occurring without plant host specialization. Unlike the IGR region, the CP-MP junction displayed a haplotype pattern that suggests a stronger host-specific adaptive evolution. This argues that the CP-MP region undergoes positive selection for adaptations to different hosts, and indicates that beneficial mutations enhancing fitness in specific hosts, rapidly increase in frequency within populations. Furthermore, such a profile of host-driven diversification may suggest that different selective pressures acting on each host are driving the evolution of EPRVs isolates that are adapted to those hosts. These findings illustrate how different EPRVs genomic regions can exhibit varying evolutionary patterns based on their roles in host adaptation processes. Similar differences between coding and non-coding regions are common in viruses (Nekoduka et al., 2015).

The distribution of pairwise differences between pairs of sequences is fundamental to understanding molecular evolutionary processes (Mousset et al., 2004). Indeed, Sequence mismatch analysis (MMA) is currently assessed to reconstruct historical demography (Grant, 2015). Viral sequences MMA can exhibit either unimodal or bimodal patterns, each indicating distinct evolutionary and demographic scenarios (Husev and Rovenchak, 2021).

In this study, the bimodal distribution within the IG region suggets that this region has experienced fewer regulatory bottlenecks followed by distinct expansion cycles despite the mixed host pattern. These two distinct demographic events are likely representing two major population phases depicting the host colonization history with a major initial colonization followed by secondary expansion and host jumps to colonize new plant species. Conversely, the CP-MP region displayed a ragged multimodal mismatch distribution suggesting a complex adaptive history characterized by multiple rounds of host-specific adaptation events. These events are accompanied by recurrent positive selection that includes several waves of beneficial and host-specific mutations.

At demographic equilibrium, the accumulation of significant divergences among lineages generally leads to a ragged, multimodal mismatch distribution (Grant, 2015). In contrast, a recent population expansion is often inferred from a smooth, unimodal distribution. However, it is important to note that statistical tests meant to evaluate the raggedness of these distributions tend to have limited sensitivity (Ramos-Onsins and Rozas, 2002). The IG region with the lowest raggedness test value supports the bimodal pattern representing whereas the CP-MP raggedness aligns with the multimodal pattern indicating a more complex, irregular demographic history with multiple overlapping events. Based on our findings, we highlight the importance of the IGR as a crucial genomic element for understanding the role of EPRVs in the dynamics of the tomato genome under environmental stress. Consequently, we selected this region for further molecular investigation into tomato genotypes response to salinity stress.

### 4.3 Management strategies in the context of salinity

Previous studies have addressed the adverse effects of salt stress on tomato genotypes currently cultivated in Tunisia (Gharsallah et al., 2016). Distinguishing among these genotypes is critical to prevent close genetic relatedness among parental lines and to enable efficient selection within segregating populations. To this end, phenotypic characterization of salt response is a clue. Accordingly, we selected two tomato varieties widely cultivated by Tunisian farmers for their valuable agronomic traits and their reported contrasting sensitivity to salinity. As a first step, salt tolerance screening was performed under controlled conditions, a widely recognized, efficient, and cost-effective approach for evaluating phenotypic responses to abiotic stress (Singh et al., 2016). Salt-treated tomato genotypes displayed variable responses to salinity stress and were classified according to visual symptoms. Leaf wilting, predominantly affecting older leaves, served as the primary criterion for grouping genotypes using a pre-established scale ranging from 1 to 4 (Chookhampaeng et al., 2007). Based on visual screening and meticulous rating of symptoms, the Cbf genotype was assigned to the mildly salt-tolerant class 2, whereas the Sabra genotype was clustered in the salt-sensitive class 3. Given the varying sensitivities to salt stress, we previously categorized 60% of local tomato genotypes into sensitive classes using either Dasgan’s or Chookhampaeng’s scale (Gharsallah et al., 2016). As these genotypes exhibited diverse responses to salt stress, with the majority falling into sensitive clusters, it is anticipated that salinity will significantly constrain tomato yields in Tunisia.

In the next phase, we transitioned from greenhouse trials to open-field experiments using the two salt-contrasting tomato genotypes with distinct susceptibility to salt stress, intending to cultivate them under innovative agronomic practices. One of the farming practices is the intercropping strategy involving tomato plants with halophyte species. Despite divergent outcomes reported across studies, it is postulated that this strategy may attenuate the tomato plant’s susceptibility to salinity stress (Jurado et al., 2024). In the present investigation, we implemented two different farming strategies alongside the control treatment (T^C^). The first involved plants grown under salt treatment (T^MC^), while the second corresponded to an intercropping system, where tomatoes were grown sequentially with halophyte crop species (T^IC^). The proximity to halophytes appears to mitigate the adverse effects of salt stress on tomato growth and maintenance, as both genotypes demonstrated recovery capacity over a three-month cultivation period. The Cbf genotype was the one that showed the better development and seemed to withstand salt stress effectively. Recently, two tomato cropping strategies were implemented corresponding to an intercropping with halophytes on moderately saline soils, and a sequential cropping where tomatoes followed halophyte cultivation (Jurado et al., 2024). Both strategies led to reduced soil salinity due to the ability of halophyte species to accumulate salt within their tissues. (Liang and Shi, 2021; Nanhapo et al., 2017; Wang et al., 2022). The reduced salt levels in the crop rhizosphere under halophyte cultivation would result in a better adaptation of the salt-sensitive plants to these extreme environments, allowing their cultivation in marginal, underutilized lands or in salt-affected agricultural soils (García-Caparros et al., 2020).

Given these reports, our T^IC^ strategy enabling tomato genotypes to recover from the adverse effects of salinity, represents a cost-effective and efficient strategy to foster production in salt-affected areas. The use of intercropping systems in agriculture is a traditional practice diffused worldwide for long time, based on the evidence of reciprocal benefits and yield increase in comparison to the monoculture in a low input system (Glaze-Corcoran et al., 2020; Maitra et al., 2021).

### 4.4 The EPRVs dynamics are shaped by farm practices

The quantification of EPRVs based on the IGR region indicated that salt stress affected the number of copies solely in the salt-sensitive genotype. In contrast, salinity did not affect the salt-tolerant genotype, regardless of the farming strategy adopted. Environmental stress is increasingly recognized as a key regulator of transposon dynamics in plants, as many TEs can become transcriptionally or transpositionally active in response to abiotic stimuli. Genome-wide studies have identified stress-responsive retrotransposons across diverse species, including *Helianthus annuus* (Mascagni et al., 2020), *Populus* (Vangelisti et al., 2019), and various conifers (Voronova et al., 2014; Fox et al., 2018). Stress also activates transposons in tissue culture and interspecific hybrids, highlighting their responsiveness to both environmental and artificial stimuli (Negi et al., 2016). These elements contribute to adaptive plasticity under fluctuating environmental conditions (Ito et al., 2022). In wheat, salinity stress has been shown to increase both the copy number and genomic insertion frequency of retrotransposons (Dilmen et al., 2023).

When coping with environmental stress, genomic instability may trigger transcription ofEPRVsfollowed by reverse transcription and integration into the genome. Indeed, environmental stressors can trigger the activation of TEs, which, once mobilized, may influence gene expression regulation within the host genome (Ito et al., 2022). These findings suggest EPRVs act as stress-responsive elements, potentially aiding adaptation or reflecting collateral activation under environmental pressure. Stress-specific associations between TEs and nearby genes were revealed in *Arabidopsis* and tomato (Deneweth et al., 2022). Once activated, these TEs were predominantly intronic and preferentially located near upregulated genes, with novel and known regulatory motifs suggesting their involvement in stress regulation. This cross-species analysis revealed that TE-driven gene regulation enables adaptation to environmental changes. The rice TE, *mPing*, was the first identified with active mobility in plants, exhibiting high transposition in the ‘Ginbouzu’ cultivar (Nakazaki et al., 2003). While most Japanese varieties like ‘Nipponbare’ harbor ~50 copies, ‘Ginbouzu’ contains over 1,000 due to active movement. *mPing* inserts preferentially near genes, particularly in promoter regions, where it can enhance responsiveness to cold and salt stress (Naito et al., 2009). A miniature inverted-repeat transposable element (MITE) integration within the regulatory region of the transcription factor gene ZmNAC111 has been linked to natural differences in drought resistance among maize varieties (Mao et al., 2015). The discovery of this TE insertion elucidates the molecular mechanisms underlying naturally occurring drought tolerance variation in maize populations. Endogenous banana streak virus, eBSV, displays several insertions across ten chromosomes in *Musa acuminata*, all of which were highly recombinant and fragmented, rendering them incapable of producing infectious viruses (Chabannes et al., 2021; Chen et al., 2024). Complementary work on eggplant genomes uncovered four distinct endogenous viral genomes alongside various EPRVs, highlighting the complexity of viral gene integration across species (Valli et al., 2023).

The presence and distribution of EPRVs within host genomes are influenced by historical viral integration events, as well as the subsequent processes that can either amplify or reduce these integrated sequences over time. Consequently, EPRVs may either remain at their original insertion sites or relocate to other genomic regions. These relocations exhibit distinct site preferences under certain conditions, whereas in other instances, they integrate randomly without specific site selection (Richert-Pöggeler et al., 2021). To regulate EPRVs, plant genomes frequently inactivate their copies through sequence degradation, rearrangement or fragmentation, preventing effective transcription of full-length viral genomes. When these endogenous pararetroviral sequences contain inverted repeats, they produce hairpin RNA structures that activate the plant’s RNA silencing machinery and small interfering RNAs production (Valli et al., 2023). This process essentially demonstrates how plants exploit integrated viral elements to potentially prevent the activation of dormant plant EPRVs and related pararetroviruses.

Another common mechanism is epigenetic silencing, which involves methylation and the action of small RNAs (sRNAs) (Staginnus et al., 2007; Schmidt et al., 2021; Noreen et al., 2007). Additionally, heterochromatin, which is typically transcriptionally inactive and exhibits low recombination rates, serves as a protective environment for EPRVs (Kent et al., 2017). Furthermore, cytosine methylation patterns and levels differ significantly across plant species, influenced by genome size and the presence of TEs (Malinowska et al., 2020). Supporting this observation, Cheng et al. (2024) reported that regions of elevated polymorphism often co-localize with CpG-rich domains undergoing epigenetic remodeling, suggesting that dynamic DNA methylation patterns may play a pivotal role in influencing the evolutionary trajectory of viral sequences.

The epigenetic status of the EPRVs’ IGR was assessed by profiling cytosine methylation patterns in symmetric (CG, CHG) and asymmetric (CHH) contexts across both salt-contrasting tomato genotypes. Salt stress imposition in a monocropping context was found to alter the dynamic methylation status of the IGR region in the salt sensitive genotype. This promoted cytosine hypermethylation mainly in the CHH and CHG motifs, providing a link between EPRVS epigenetic regulation and the plant’s response to salt stress. This aligns with findings in other crops, where hypermethylation has been linked to reduced stress tolerance (Wu et al., 2024). The stable maintenance of CHG methylation and subtle alterations in CHH methylation patterns observed in the salt-tolerant genotype may indicate enhanced regulation of genes involved in osmo-protection and ion homeostasis. Similar methylation patterns have been identified in stress-resilient genotypes, which actively maintain specific methylation states to trigger stress-responsive gene expression (Kumar and Mohapatra, 2021). The IGR is likely a differentially methylated region (DMRs) despite the fact that CGH methylation remained unchanged across T^MC^ and T^IC^ strategies for both genotypes. These findings highlight the evolutionary constraint on EPRVs to remain transcriptionally silent, reflected by CGH methylation stability, while also revealing intrinsic variability and epigenetic plasticity in silencing intensity, potentially enabling adaptive responses to environmental conditions.

## 5 Conclusion

Within tomato, the EPRVs’ occurrence mediates a fine balance between preserving host genome stability and allowing for copy number variability. These changes, along with epigenetic regulation through the level of cytosine methylation, help alleviate salinity stress in tomato across both monoculture and intercropping systems. Based on our research findings, halophyte intercropping with tomato emerges as a promising agronomic strategy for enhancing vegetable production in saline-affected, marginal arid and semi-arid environments. Conducting field trials in these saline environments is recommended to validate the phytodesalination potential of halophytes under natural conditions.

## Data Availability

The original contributions presented in the study are included in the article/**Supplementary Material**. Further inquiries can be directed to the corresponding author.
