# Supplementary material for: Genetic diversity and evolution of endogenous pararetroviruses across Solanaceae: How farming systems drive dynamic tomato EPRVS changes under salt stress
Source: Front Plant Sci. 2026 Jan 30;17:1702837. doi: 10.3389/fpls.2026.1702837 (PMC12904150; doi:10.3389/fpls.2026.1702837)
Supplement: Supplementary file 1 [file DataSheet1.docx]

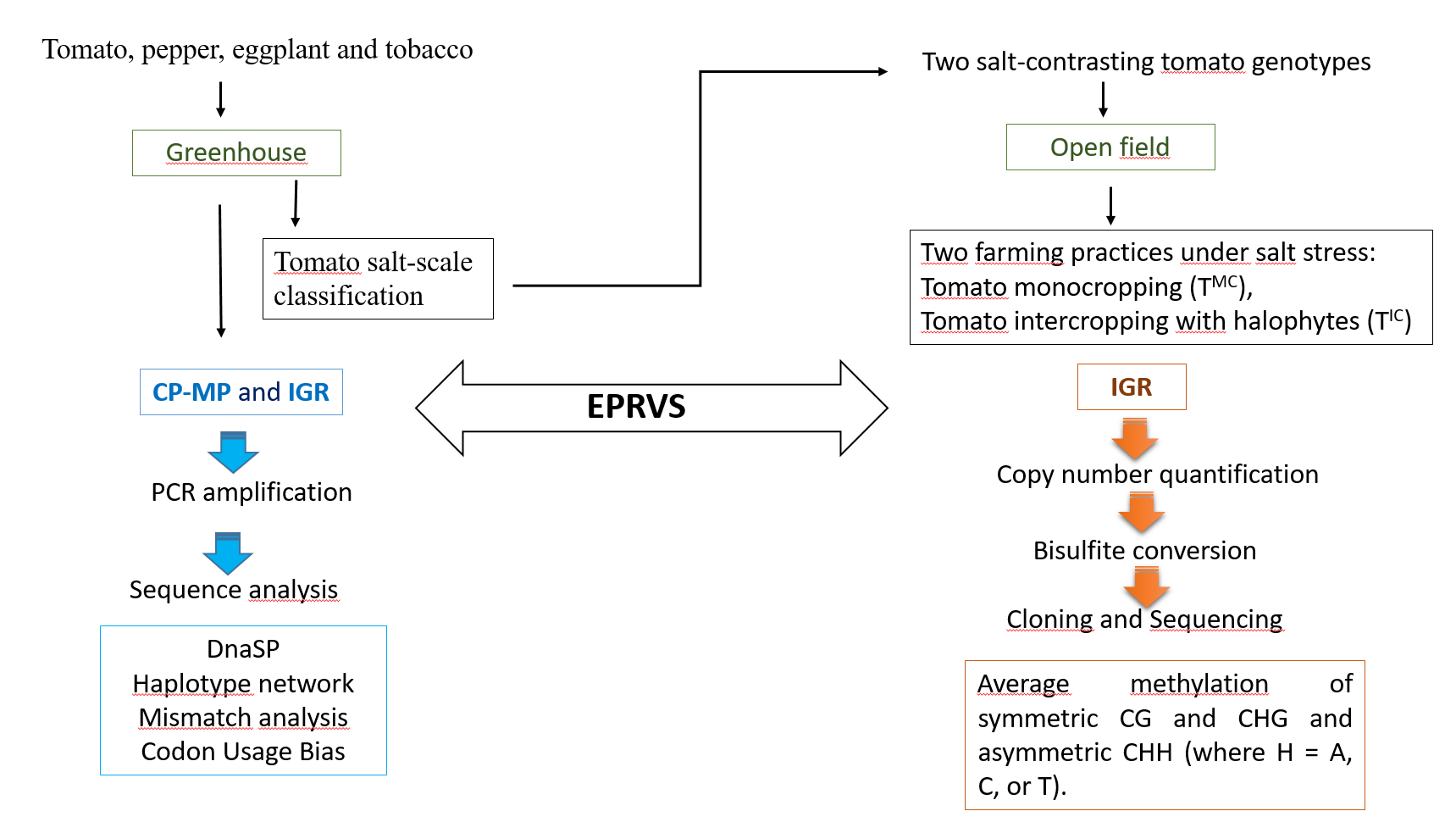


Figure S1 : Diagram illustrating the workflow implemented in this work.


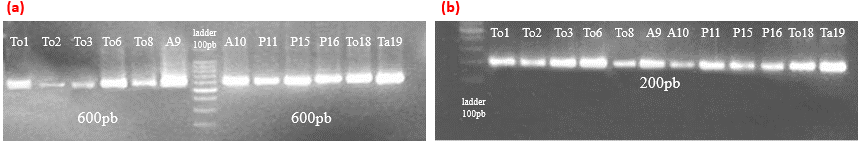


Figure S2 : PCR produts corresponding to (a) CP-MP junction and (b) IGR region of EPRVs in Solanaceous genomes (To: tomato ; A: Eggplant ; P: pepper ; Ta: tobacco).

**Table S1. Codon Usage of the coding CP-MP region among the *solanaceae* species**

| UUU(F)   3.7(1.91) | UCU(S)   0.7(0.46) | UAU(Y)   7.4(1.32) | UGU(C)   1.8(1.33) |
| --- | --- | --- | --- |
| UUC(F)   0.2(0.09) | UCC(S)   0.8(0.57) | UAC(Y)   3.8(0.68) | UGC(C)   0.9(0.67) |
| UUA(L)   3.9(2.19) | UCA(S)   1.7(1.14) | UAA(*)   4.8(0.99) | UGA(*)   6.0(1.25) |
| UUG(L)   2.1(1.16) | UCG(S)   0.0(**0.00**) | UAG(*)   3.7(0.76) | UGG(W)   0.4(1.00) |
| CUU(L)   1.2(0.65) | CCU(P)   1.1(0.98) | CAU(H)   1.7(1.25) | CGU(R)   0.2(0.08) |
| CUC(L)   0.2(0.09) | CCC(P)   0.8(0.75) | CAC(H)   1.0(0.75) | CGC(R)   0.1(0.04) |
| CUA(L)   1.8(0.98) | CCA(P)   2.4(2.19) | CAA(Q)   3.3(1.11) | CGA(R)   1.5(0.73) |
| CUG(L)   1.7(0.93) | CCG(P)   0.1(0.08) | CAG(Q)   2.6(0.89) | CGG(R)   0.1(0.04) |
| AUU(I)   4.2(0.57) | ACU(T)   1.8(0.80) | AAU(N)   9.0(1.36) | AGU(S)   4.3(2.91) |
| AUC(I)   2.7(0.37) | ACC(T)   2.8(1.20) | AAC(N)   4.3(0.64) | AGC(S)   1.3(0.91) |
| AUA(I)  14.9(2.06) | ACA(T)   3.7(1.60) | AAA(K)  14.6(1.42) | AGA(R)   8.3(**4.05**) |
| AUG(M)   4.6(1.00) | ACG(T)   0.9(0.40) | AAG(K)   6.0(0.58) | AGG(R)   2.2(1.05) |
| GUU(V)   0.8(0.62) | GCU(A)   0.8(1.29) | GAU(D)   7.0(1.53) | GGU(G)   0.7(0.76) |
| GUC(V)   0.3(0.28) | GCC(A)   0.3(0.52) | GAC(D)   2.2(0.47) | GGC(G)   0.0(**0.00**) |
| GUA(V)   3.3(2.69) | GCA(A)   1.1(1.68) | GAA(E)  11.4(1.69) | GGA(G)   2.8(3.24) |
| GUG(V)   0.5(0.41) | GCG(A)   0.3(0.52) | GAG(E)   2.1(0.31) | GGG(G)   0.0(**0.00**) |

Relative synonymous codon usage is given in parentheses following the codon frequency
